# A comparative study: impact of chemical and biological fungicides on soil bacterial communities

**DOI:** 10.1186/s40793-025-00713-6

**Published:** 2025-04-29

**Authors:** Setu Bazie Tagele, Emma W. Gachomo

**Affiliations:** Department of Microbiology and Plant Pathology, University of California Riverside, Riverside, CA 92507 USA

**Keywords:** Bacterial diversity, Eco-plate, Illumina miseq, Ridomil, SoilGard™ 12G

## Abstract

Soil microbial communities play key roles in agroecosystems, particularly in processes like organic matter decomposition and nutrient cycling. However, human activities can negatively impact their community structure and, consequently, soil function. SoilGard and Ridomil are effective methods for controlling carrot cavity spots caused by *Pythium* spp., but their effects on bacterial taxonomic and metabolic function shifts are not well understood. This study aims to investigate the comparative impact of the chemical fungicide Ridomil and the biological fungicide SoilGard on the bacterial communities in soils cultivated with carrots. Our results showed that both SoilGard and Ridomil significantly impacted soil bacterial diversity, but their effects were distinct and time-dependent. Ridomil had an immediate negative effect on soil bacterial diversity two weeks after treatment, whereas SoilGard was initially less disruptive but showed delayed negative consequences 12 weeks after treatment, particularly when combined with *Pythium* inoculation. Ridomil treatment led to an increase in Proteobacteria, especially the *Pseudomonas* population, as confirmed by both MiSeq and qPCR data. In contrast, SoilGard depleted the Mycobacterium population at 12 weeks after treatment. Furthermore, the results of community-level physiological profiling using Biolog Ecoplates showed significant differences in substrate-level diversity between Ridomil and SoilGard-treated samples, indicating a shift in the metabolic activity of bacterial communities. Ridomil-treated samples showed the lowest metabolic activity of bacterial communities, based on the diversity and richness of carbon source utilization, compared to control. Overall, this research highlights the distinct and time-dependent effects of biological and chemical fungicides on soil bacterial communities when applied at recommended doses.

**Supplementary Information:**

The online version contains supplementary material available at 10.1186/s40793-025-00713-6.

## Introduction

Soil is a living entity that supports life and soil functions as a natural resource [1–3]. Soil microbial communities are considered integral to soil fertility, crop health, and productivity, playing key roles in agroecosystems [2, 4]. These microbes form the first line of defence against soil-borne pathogens through a variety of mechanisms [5]. A wide range of beneficial soil microbes recruited by plants contribute to pathogen suppression by producing antimicrobial compounds which directly target soil-borne pathogens [6, 7]. In addition, these microbes help protect plants by competing for space and resources, making it difficult for the pathogens to establish themselves in the rhizosphere [8]. Other mechanisms involve the indirect activation of plant immune responses, triggered by beneficial microbes to protect against pathogen attack [3, 7]. Furthermore, soil microbes enhance plant tolerance to environmental stresses by regulating phytohormones and reducing ethylene levels [1–3]. Soil microbial communities are also crucial for nutrient cycling, including nitrogen fixation and increasing phosphorus availability through mobilization and immobilization. They also enhance plant growth via phytostimulation [1, 2, 5]. They degrade various soil pollutants, and maintain soil structure by influencing soil aggregation and porosity through production of extracellular polymeric substances (EPS), organic matter decomposition, bacterial filaments, and fungal hyphae [1, 2].

Previous reports have shown that chemical and microbial fungicides significantly impact non-target soil bacterial communities [9]. Studies have demonstrated a reduction in the taxonomic diversity of the bacterial population following fungicides, with a noted prevalence of Proteobacteria, including *Pseudomonas*, suggesting their fungicide-tolerance nature. Some bacterial populations are negatively impacted by the fungicides due to direct toxicity [10] and bacterial tolerance to fungicide exposure is thought to be variable [11, 12]. Bacteria interact with fungi, and any impact on fungal populations by fungicides indirectly affects bacterial populations [11, 13]. Furthermore, fungicides affect not only taxonomic diversity but also functional diversity. For instance, Carbendazim fungicide increased bacterial metabolic activities, and bacterial species utilized Carbendazim as a carbon source [12]. Similarly, Ridomil application led to higher bacterial activity, suggesting that Ridomil could serve as an energy source for bacterial communities by providing dead fungi as a substrate for bacterial communities [14]. These non-target negative effects have significant implications for soil quality, which is essential for a sustainable agroecosystem.

Ridomil and SoilGard are chemical and biological fungicides commonly used to control soil-borne diseases, including carrot cavity spot (CCS) caused by *Pythium* spp [13, 15]. Our studies have revealed significant impacts of Ridomil and SoilGard on fungal community dynamics [13, 15]. Although some reports showing changes in bacterial taxonomic profiling following application of Ridomil exist, they are based on the position of amplification bands rather than sequencing results [16]. Previous studies that reported Ridomil’s effects on soil physiological properties and microbial biomass [17], did not show its impact on bacterial taxonomy and associated functional diversities. There is growing interest in using microbial biofungicides, including *Trichoderma*-based biocontrol agents, for controlling soil-borne diseases like *Pythium* spp., as biofungicides are considered more sustainable for agroecosystems [18, 19]. Assessing the impacts of chemicals and biofungicides on soil bacterial communities is crucial to improving fungicide regulation and developing novel strategies with minimal impact on soil biology, thus ensuring a sustainable approach to plant disease management [9].

This study aimed to investigate the comparative impact of chemical fungicide Ridomil and the biological fungicide SoilGard on the bacterial communities in soil cultivated with carrots. We hypothesize that the recommended rates of SoilGard and Ridomil have distinct impacts on bacterial taxonomic diversity and metabolic activities due to their variable modes of action and chemistries. Furthermore, given that the active ingredient of SoilGard is *Trichoderma*, a soil borne microorganism, we hypothesize that SoilGard would have less negative impacts on soil bacterial diversity compared to Ridomil.

## Materials and methods

### Greenhouse pot experiment and sampling

Carrot seeds of the Crispy Cut variety were grown in UC Soil Mix III [20] in 3 L pots under greenhouse conditions. The pots were hand irrigated two times per week with 1% Peters Mix fertilizer (Peter’s 21-5-20 Excel Multi-Purpose, Scotts, USA) during the first month and placed under drip irrigation for the rest of the growing season. The insecticides Tristar (by NuFarm) and Captiva Prime (by Gowan) were each sprayed once during the season to control thrips and whiteflies. One month after planting, six isolates from three *Pythium* species, known to cause carrot cavity spot (CCS), were inoculated into the soil at equal concentrations (667 cfu g^− 1^) to form a total of 4000 cfu g^− 1^. One month after planting was chosen because that is the time the carrot tap root starts expanding based on our experience. The *Pythium* species used were *Pythium irregulare*,* Pythium ultimum*, and *Pythium sulcatum*. The *Pythium* inoculum preparation and inoculation were performed as described in our previous reports [13]. On the same day, Ridomil (Ridomil Gold SL, 45.3% a.i. mefenoxam, Syngenta USA) and SoilGard (Certis USA, a.i. *Tichoderma virens* strain GL-21) were applied to the soil at manufacturer recommended rates: 0.499 µL L^− 1^ and 0.9 g L^− 1^, respectively. These treatments were also applied to *Pythium* non-inoculated pots. Pots that received no Ridomil or SoilGard treatments served as untreated controls. The experiment was laid out in a completely randomized design with three replications (pots) per treatment. The treatment combinations in the experiment were as follows: Treatment 1: Ridomil plus *Pythium*, Treatment 2: Ridomil without *Pythium*, Treatment 3: SoilGard plus *Pythium*, Treatment 4: SoilGard without *Pythium*, Treatment 5: Control plus *Pythium*, and Treatment 6: Control without *Pythium*. In the control, water was used instead of the fungicide. Pots were watered through a drip irrigation system and fertilized twice in the first month before the treatments were applied.

Soil sample collection was performed using an open-ended 1-centimeter diameter syringe at 2 weeks and 12 weeks (ST1 and ST2, respectively) after treatment application. Two weeks after treatment is an early time point soon after treatment and 12 weeks after treatment coincide with harvesting time. Samples were taken by twisting and pushing the syringe to penetrate up to a depth of 5 cm. The bottom 3 cm of soil was accessed by pushing the plunger. Three samples were collected per pot and combined to form a single composite sample for each pot. There were three replications (three composite samples) for each treatment.

### DNA extraction and illumina miseq library Preparation

DNA was extracted from 250 mg soil samples using DNeasy Powersoil kit (Qiagen, Valencia, CA, USA) according to the manufacturer’s instructions. The quality of the extracted DNA was checked using an Implen Nanophotometer (Implen, Westlake Village, CA, USA). The changes in soil bacterial communities following the treatments in soil were characterized by Illumina amplicon sequencing of the bacterial 16 S ribosomal RNA gene. The Illumina MiSeq library was prepared by amplifying the highly variable V5-V6 region. A two-stage PCR process was used to amplify the target region. In the first stage PCR (amplicon PCR), the target region was amplified using 0.2-µM primers, the Phusion high-fidelity PCR master mix with HF buffer (Thermo Scientific), and 1 µL of extracted DNA. The PCR conditions were as follows: initial denaturation at 98 °C for 30s, followed by 23 cycles of 98 °C for 10s, annealing at 56.5 °C for 30s, and 72 °C extension for 30s, with a final elongation step at 72 °C for 5 min. The size of the PCR products and amplification efficiency were checked using gel electrophoresis, followed by a clean-up step using AMPure XP beads and freshly prepared 80% ethanol.

In the second stage PCR (index PCR), the cleaned PCR products from the first stage were used as templates to attach the barcodes and illumina sequencing adaptors. The following PCR conditions were used: initial denaturation at 98 °C for 30s, followed by 6 cycles of 98 °C for 10s, annealing at 65 °C for 30s, and 72 °C extension for 30s, with a final elongation step at 72 °C for 5 min. The second stage PCR product was cleaned using AMPure XP beads and its concentration was quantified using the Qubit^®^ 2.0 Fluorometer (Life Technologies, Carlsbad, CA, USA). Cleaned amplified products were pooled in equal molar concentrations of 5 nM. The size and concentration of the final library was verified using a 2100 Bioanalyzer (Agilent) and Illumina sequencing was carried out at the UCR genomics core facility.

### Quantitative PCR analysis

The effects of SoilGard and Ridomil on the abundance of total bacteria, *Pseudomonas* spp, total fungi, *Trichoderma* spp., and *Pythium* spp. were assessed using quantitative PCR (qPCR) with the Maxima SYBR green/ROX qPCR master mix (2x) (Thermo Fisher Scientific, Waltham, MA, USA) on a Bio-Rad CFX Duet Real-Time PCR System (Bio-Rad Laboratories, Inc., Hercules, CA, USA). DNA extracted from soil samples, previously utilized for Illumina sequencing (see Sect. 2.2 above), was used in this experiment.

#### Abundance of total Bacteria and *Pseudomonas* spp

Universal primers targeting the V4 hyper-variable of the 16 S rRNA gene were employed: 515 F and 806R [21, 22] (Table S1). Each PCR mixture reaction (25 µL) contained 5 ng of template DNA, 12.5 µL of Maxima SYBR green/ROX qPCR master mix, 0.3 µM of each primer, and 10 µL of nuclease-free water. The PCR conditions were as follows: initial DNA denaturation at 95 °C for 3 min, followed by 40 cycles of 95 °C for 30 s (denaturation), 55 °C for 30 s (annealing), and 72 °C for 30 s (extension). Another PCR was performed using *Pseudomonas*-specific primers: Pse435F and Pse686R (Bergmark et al. 2012) (Table S1). The PCR mixtures and conditions for *Pseudomonas* were similar to those used for the 16s rRNA gene, except for the annealing temperature, which was set to 60 °C.

The PCR products were subjected to gel electrophoresis on a 1% (w/v) gel for cleaning. The gel fragment containing the expected band size of the gene of interest was excised and purified using GeneJET PCR Purification Kit (Thermo Fisher Scientific, Lithuania) according to the manufacturer’s instructions. The concentration of the extracted DNA was then quantified using the Qubit^®^ 2.0 Fluorometer (Life Technologies, Carlsbad, CA, USA). A serial dilution, ranging from 10^1^ to 10^6^ of the PCR products for each gene of interest, was prepared separately to quantify total bacteria and *Pseudomonas*. Melt curve analysis was performed to verify the specific amplification of the target sequences in each assay. The decrease in fluorescence signal was recorded as the temperature was increased from 60 °C to 95 °C in 0.5 °C increments (Fig. S1). The gene copy number per gram of soil for all samples was calculated based on the quantification cycle (Cq) values of the standards and their corresponding gene copy numbers. This calculation took into account the amount of soil used for DNA extraction, the elution volume, the DNA concentration, and the length of the PCR product. The correlation coefficient (R^2^) values, derived from the standard curves for total bacteria and *Pseudomonas*, demonstrated high linearity: 0.9913 and 0.9824, respectively. All qPCR assays had three biological replicates and two technical replicates.

#### Abundance of total fungi, *Trichoderma* spp., and *Pythium* spp

To determine absolute abundance of total fungi, *Trichoderma* species, *Pythium* species, *Pythium irregulare*, and *Pythium ultimum* in the soil samples from each treatment, the same DNA extracted for the Illumina sequencing was used as a template. The fungal 18s rRNA gene was amplified using the universal primer set FR1 / FF390 to quantify the total fungi [23, 24]. For *Trichoderma* and *Pythium* quantification, *Trichoderma* specific tef1 genes were amplified [25] and the pyth_f/pyth_r2 primers that target ITS regions specific to *Pythium* spp [26]. were used (Table S1). In addition, the IRR3cF/IRR3R primers specific to *P. irregulare* [27] and the ULT1F/ULT4F primers specific to *P. ultimum* [27] were employed (Table S1) [25].

Genomic DNA was extracted from pure cultures of *Trichoderma virens*, *P. irregulare*, and *P. ultimum* using the DNeasy Powersoil kit (Qiagen, Valencia, CA, USA) according to the manufacturer’s instructions. The cleaned PCR products were used as standards for absolute quantification. The PCR conditions for each fungal and *Pythium* species were as described in Sect. 2.3.1 above except for the annealing temperatures (Table S1). Serial dilution, melt curve analysis (Fig. S1) and gene copy number calculations were carried out as described in Sect. 2.3.1. The correlation coefficient (R^2^) values for total fungi, *Trichoderma*, *P. irregulare*, *P. ultimum* and *Pythium* species were ≥ 0.98.

### Effect of Ridomil on the growth of *Pseudomonas* species

The impact of Ridomil at various concentrations on the growth of two *Pseudomonas* species, *P. palleroniana* B2020 and *P. protegens* B59979, was assessed using a 96-well plate assay. The *Pseudomonas* species were obtained from the USDA, ARS, National Center for Agricultural Utilization Research (NCAUR), located in Peoria, IL. The final Ridomil concentrations in growth media were as follows: 0.25, 0.5, 1, 2, 4, and 8 µl/L. Initially, a single colony from a pure culture was grown in 10 mL of LB broth for 48 h. The bacterial culture was then diluted to an OD600 value of 0.1 before adding it to the wells. A specific concentration of Ridomil was added to 8 wells in a column of the 96-well plate, except the control wells which contained water instead of Ridomil. The plates were incubated at 30 °C, and OD values were recorded at various incubation times until the growth curve reached the death phase, 48 h post-incubation.

### Metabolic profiling of bacterial communities in the soil samples

The metabolic activities of bacterial communities in the different soil samples were determined using Biolog Ecoplate (Biolog, Inc., Hayward, California, USA), which contains 31 distinct carbon sources. Fresh soil samples were collected fromthree spots in each pot (replicate) and combined to make one representative sample for each replicate. There were three replicates (pots) per treatment. For each replicate, a 1-gram soil sample was added to a 99 ml sterile sodium chloride (0.85%) solution, and vortexed for 10 min. The suspension was allowed to settle for 10 min, and 100 µL of the suspension was loaded into an Ecoplate well in a laminar flow hood. The plates were incubated at 30 °C for six days in the dark and the color change in the wells was monitored by measuring the absorbance at 590 nm using a SpectraMax iD5 Multi-Mode microplate reader (Molecular Devices, San Jose, CA, USA).

Further metabolic analysis was performed by categorizing the carbon sources into different groups: amino acids, amines and amides, carbohydrates, carboxylic acids, and polymers according to [28]. Heat map and line graphs were used to display the differences in carbon sources utilization in different samples at various incubation periods.

### Analysis of the soil carbon and nitrogen content

To determine the effects of the treatments on soil carbon (C) and nitrogen (N), soil samples were dried overnight at 105 °C, ground and sieved through a 100-micron mesh sieve. One hundred milligrams of the soil was weighed into aluminum capsules (CE Elantech, NJ USA, part # 252-080-00), that were then sealed by twisting the foil with tweezers. The samples were stored in a desiccator prior to analysis. The C and N contents were analysed by flash-combustion/oxidation using a Thermo-Finnigan Flash EA1112 elemental analyzer at the Environmental Sciences Research Laboratory (University of California Riverside). The values obtained were used to calculate the C/N ratio.

### Bioinformatics

Demultiplexed amplicon DNA sequence data files were imported into QIIME 2 [29] using the ‘qiime tools import’ plugin and converted into.qza file format. The ‘qiime demux summarize’ plugin was used to visualize and assess the sequence quality. Sequences with quality scores above 30 were retained. Primer sequences were trimmed using–p-trim-left and–p-trim-right parameters, and sequences were truncated at 125 bases using the ‘qiime dada2 denoise-single’ plugin [30]. Singletons and chimeras were filtered out. The qiime feature-table filter-samples plugin showed that the sequence counts across samples were over 10,000. Low-frequency features, present in less than 0.1% of at least two samples, as well as contaminants and unclassified features, were filtered out using the ‘qiime feature-table filter-features’ plugin. Taxonomy assignment was performed using the pre-trained scikit-learn naive Bayes classifier trained on Silva 138 99% OTUs full-length sequences from the Silva reference database via the ‘qiime feature-classifier classify-sklearn’ plugin [31]. A rooted phylogenetic tree was generated using the command: ‘qiime fragment-insertion sepp’. An alpha-rarefaction curve was constructed to assess the species richness and ensure uniform sampling depth of 14,582 reads per sample across samples (Fig. S2). Diversity metrics were determined using ‘qiime diversity core-metrics-phylogenetic’, and statistical significance tests were performed using ‘qiime diversity alpha-group-signiificance’ and ‘qiime diversity beta-group-signiificance’ Qiime 2 plugins. The final rarefied dataset in.qza format was exported using ‘qiime tools export’ for downstream analysis in R.

### Statistical data analysis

Statistical data analysis and visualization were performed using R free software (version 4.2.3). Excel files were imported with the xlsx R package [32]. Analysis of variance for alpha diversity indices was performed using the agricolae package [33] with Duncan’s multiple range test and visualized with ggplot2 package [34]. The dplyr package was used for sorting the data for analysis and visualization [34]. Beta diversity analysis to evaluate bacterial community composition differences among samples (PERMANOVA) was performed using the vegan package [35]. Linear discriminatory analysis (LDA) effect size (LEfSe) analysis was conducted with the LEfSe package in R [36]. An LDA score over 3 was used to visualize the differentially abundant taxa across samples. Sankey diagrams showing the relative abundance of genera across samples were constructed using the ggalluvial R package [37]. Average of the three replications per sample was used for visualization. Sorting and subsetting Biolog Ecoplate data under different carbon sources categories were performed using the dplyr package and the data were analyzed with agricolae package and visualized with ggplot2. The ComplexHeatmap package was employed to construct the hierarchical clustered heat map of carbon sources utilization at different incubation periods [38].

## Results

### Ridomil had transient impact on bacterial diversity

**Fig. 1:**
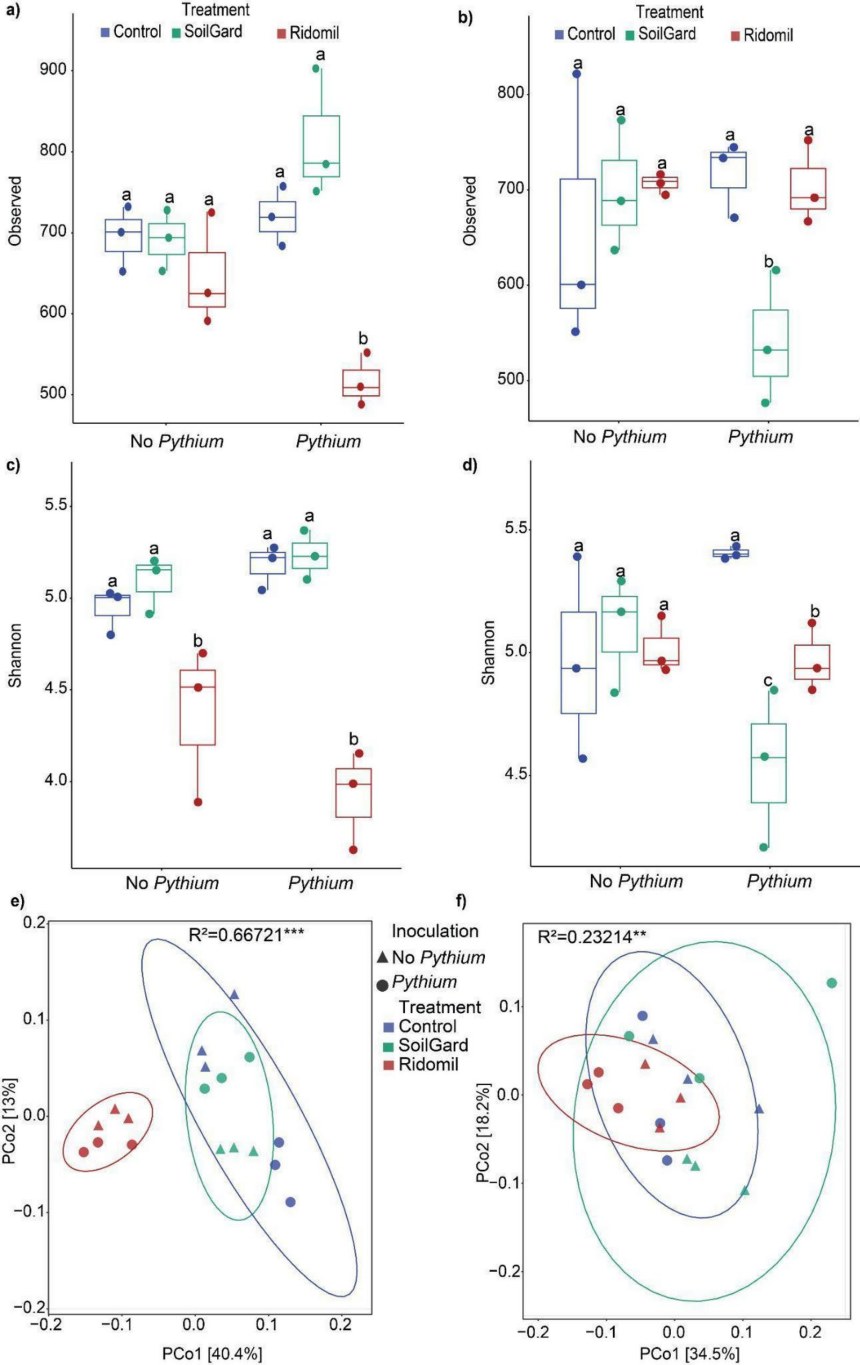
SoilGard and Ridomil exhibit distinct effects on bacterial diversity under carrot cultivation. Observed species (**a** and **b**), Shannon diversity (**c** and **d**). Figure panels **a**, **c** and **e** represent data at ST1, while figure panels **b**, **d** and **f** represent data at ST2. Principal component analysis based on weighted UniFrac distances illustrates the distribution pattern of bacterial communities in different samples at ST1 (**e**) and ST2 (**f**). ** and *** denotes PERMANOVA significant differences at *p* < 0.01 and 0.001, respectively. ST1 and ST2 represent soil sampling times 2 and 12 weeks after treatment application, respectively

In our study, alpha diversity indices, including Observed and Shannon, indicated that the influence of Ridomil and SoilGard on bacterial diversity varied with sampling time (Fig. 1a-d). Soil treated with Ridomil under carrot cultivation exhibited significantly (*p* < 0.05) lower bacterial diversity compared to the untreated control at 2 weeks post-treatment (first soil sampling time, ST1), but SoilGard showed no significant effects during this period (Fig. 1a. 1c). However, by 12 weeks after treatment (second soil sampling time, ST2), SoilGard in combination with *Pythium* inoculation, significantly reduced bacterial diversity compared to the untreated control (Fig. 1b. 1d). The observed reduction in diversity following SoilGard application was significant in *Pythium-*inoculated soil, but not after treatment with SoilGard or *Pythium* alone (Fig. 1a-d). The lowest Observed species and Shannon diversity were recorded after Ridomil plus *Pythium* treatment at ST1, and SoilGard plus *Pythium* treatment at ST2 (Fig. 1a-d).

The β-diversity analysis, based on weighted UniFrac distances, revealed a significant shift in the bacterial community structure following treatment application (PERMANOVA, *p* < 0.01) (Fig. 1e, f). In Ridomil-treated samples, regardless of *Pythium* inoculation, formed distinct clusters separate from the other treatments across PCo1, accounting for 40.4% variation at ST1.

### Bacterial community structure shifts following application of Ridomil and SoilGard

**Fig. 2:**
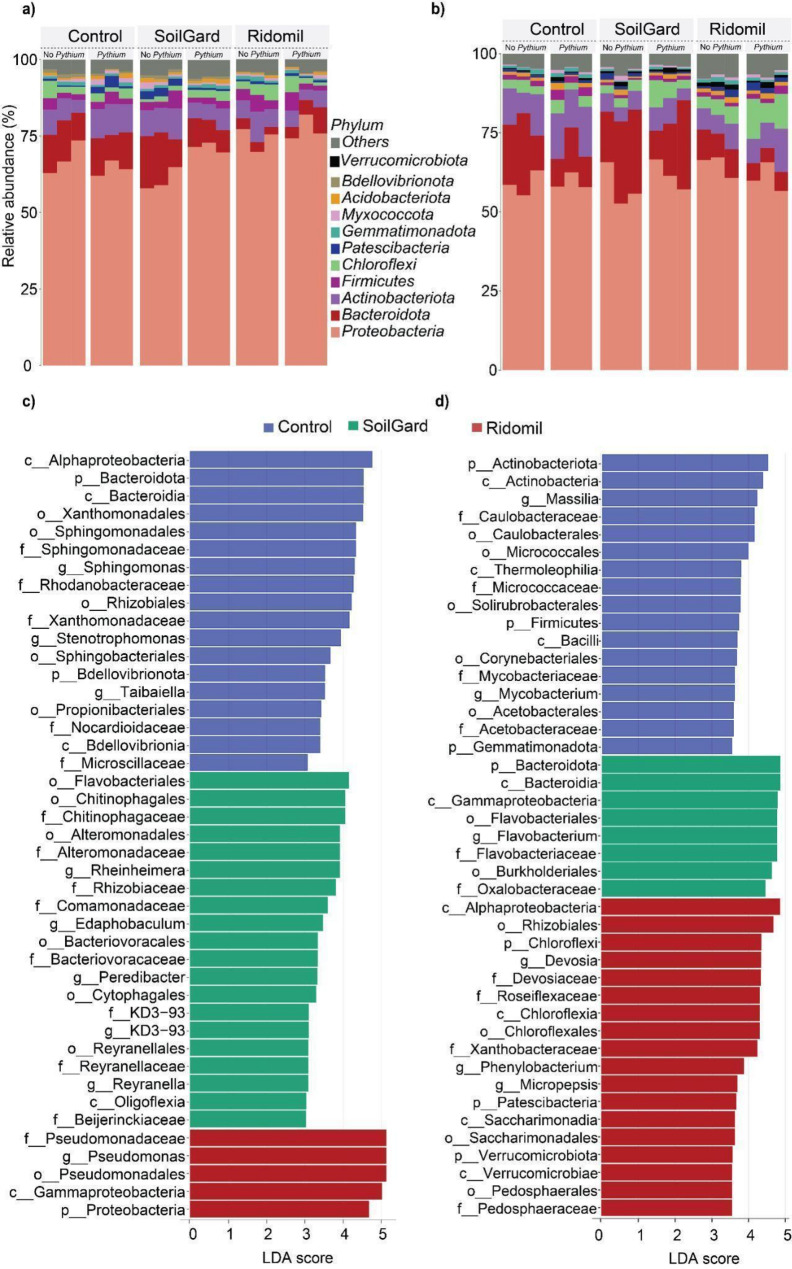
Response of Soil bacterial communities to SoilGard and Ridomil application under carrot cultivation. Relative abundance at the phylum level at ST1 (**a**) and ST2 (**b**). Differentially abundant taxa following biofungicide and fungicides application were identified using linear discriminatory analysis (LDA) effect size (LEfSe) analysis with LDA > 3 (*p* < 0.05) at ST1 (**c**) and ST2 (**d**). ST1 and ST2 represent soil sampling periods of 2 and 12 weeks after treatment application, respectively

**Fig. 3:**
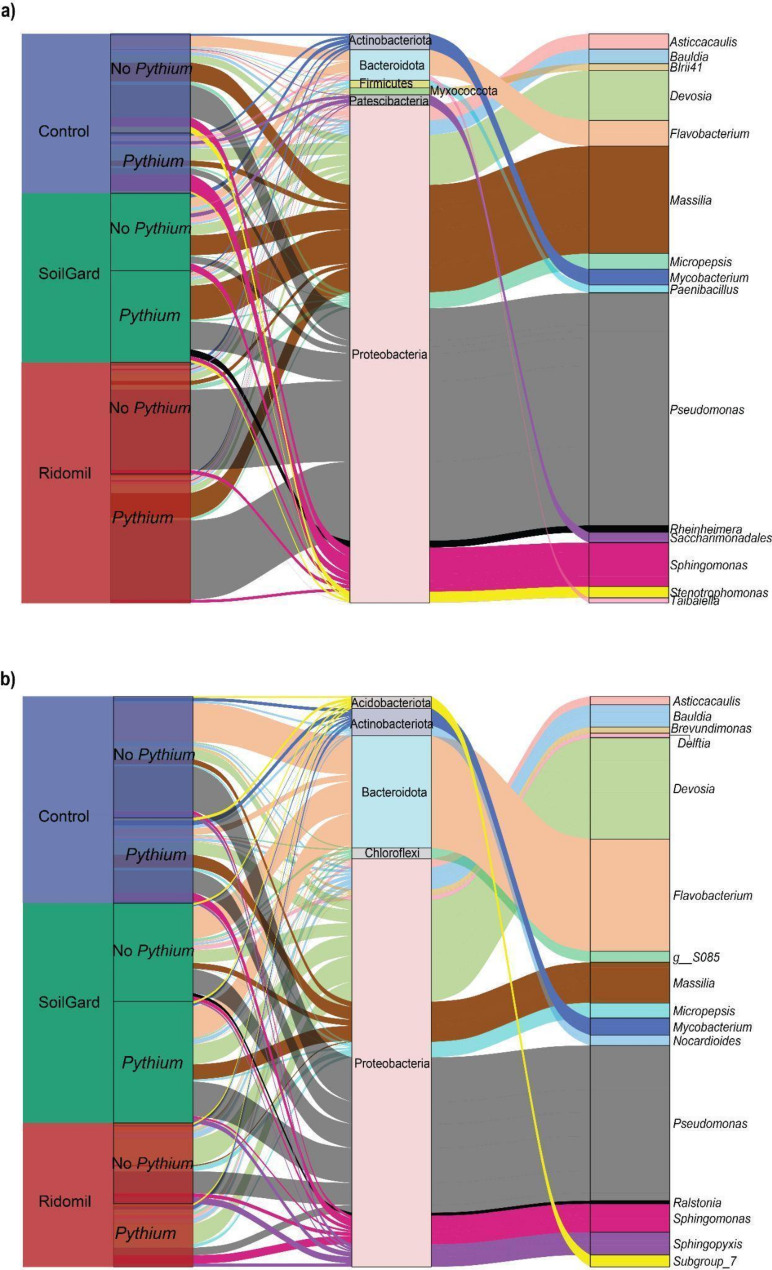
Impacts of SoilGard and Ridomil on genus-level relative abundance in soil cultivated with carrot. Sankey diagrams displaying relative abundance at the genus level at ST1 (**a**) and ST2 (**b**). ST1 and ST2 represent soil sampling periods of 2 and 12 weeks after treatment application, respectively

The taxonomic composition of bacteria communities at the phylum, class, and genus level are depicted in Figs. 2 and 3, and Tables S2-S2. The bacterial community at the phylum level was predominantly Proteobacteria across all samples at both sampling times (Fig. 2a, b) and its relative abundance was notably higher at ST1 compared to ST2 in all treatments. At ST1, the relative abundance of Proteobacteria significantly increased (*p* < 0.01) with Ridomil application, with or without *Pythium* inoculation, compared to the untreated control, while that of Bacteroidota significantly reduced (*p* < 0.01) (Fig. 2a, Table S2). However, the effect of Ridomil on the Proteobacteria population at ST2 was not statistically significant (*p* = 0.557) (Table S2). In addition, application of Ridomil significantly reduced the population of Bdellovibrionota at ST2 compared to the untreated control (Table S2). Interestingly, the *Pythium* inoculation together with Ridomil or SoilGard significantly reduced the Actinobacteriota population at ST1, but inoculation of *Pythium* alone significantly increased the relative abundance of the same phylum (Table S2). Chloroflexi and Patescibacteria populations were significantly increased by treatment with Ridomil and *Pythium* inoculation at ST2, while SoilGard showed no effect on these bacterial populations at both sampling time points (Table S2).

At the class level, Gammaproteobacteria and Alphaproteobacteria were the most dominant and significantly enriched classes in Ridomil-treated soil at ST1 and ST2, respectively, compared to the untreated control (Table S3). Bacteroidia was highly depleted in Ridomil-treated soil at ST1 (Table S3). In the *Pythium*-inoculated soil, Bacilli were highly reduced by the application of both Ridomil and SoilGard treatments at ST2, but these effects were not observed in the *Pythium* non-inoculated group (Table S3). At the genus level, Ridomil treatment significantly enriched *Pseudomonas* spp. at ST1 but not at ST2, while *Devosia* spp. increased at ST2 (Table S4, Fig. 3a, b). *Flavobacterium* population was significantly depleted in Ridomil-treated soil at ST1 and ST2, but SoilGard had no significant impact on this genus compared to the untreated control at ST2. However, SoilGard significantly reduced the *Mycobacterium* population at ST2 (Table S4, Fig. 3a, b). In addition, in *Pythium*-inoculated soils, *Sphingomonas* was significantly (*p* < 0.05) negatively affected by both Ridomil and SoilGard at ST1 (Table S4, Fig. 3a, b). The differential abundant analysis using LEfSe analysis also revealed that Proteobacteria, Gammaproteobacteria, and *Pseudomonas* were differentially more abundant with Ridomil treatment at ST1, while Alphaproteobacteria and *Devosia* were highly enriched in the same treatment at ST2 (Fig. 2c, d).

### *Pseudomonas* spp. abundance by qPCR and in vitro growth assay

**Fig. 4:**
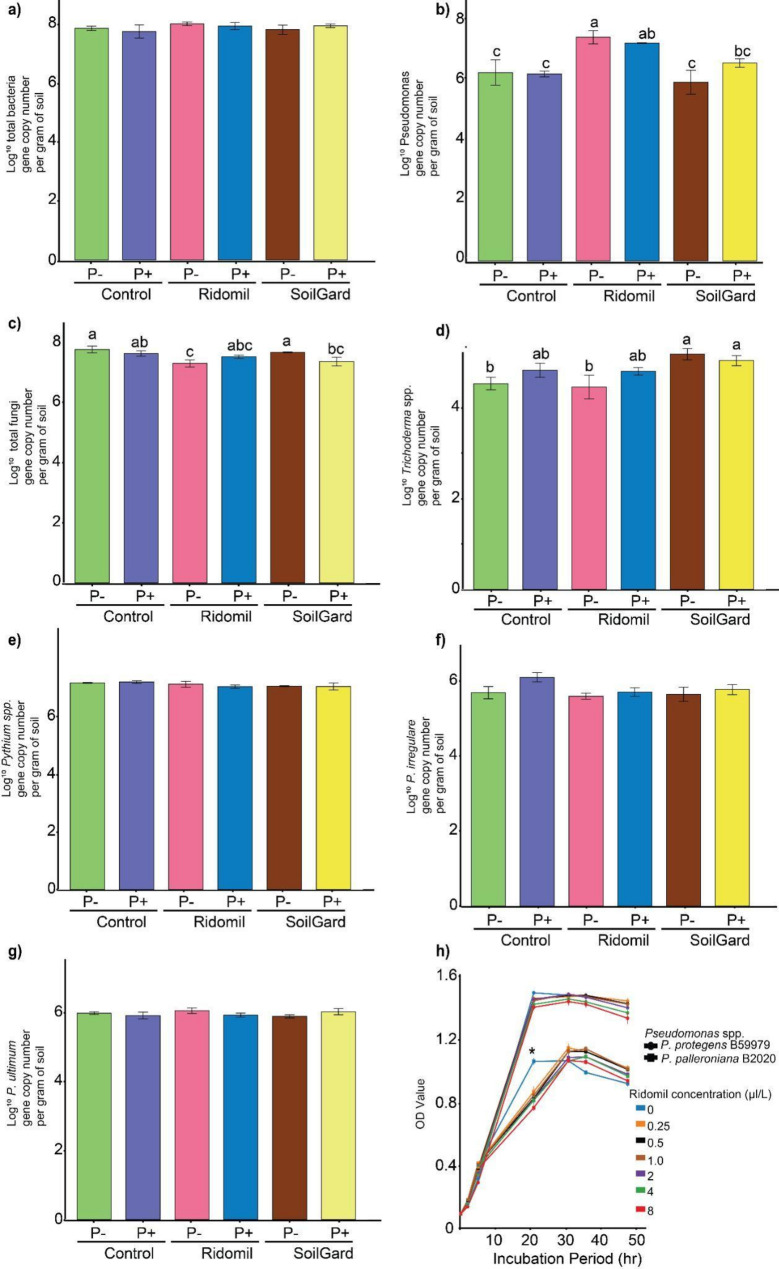
Quantification of Total Bacteria, Total Fungi, and Specific Species. Gene copy number of total bacteria (**a**), *Pseudomonas* (**b**), total fungi (**c**), *Trichoderma* (**d**), *Pythium* species (**e**), *P. irregulare* (**f**) and *P. ultimum* (**g**). The impact of different concentrations of Ridomil on the growth of two *Pseudomonas* species, as determined by OD value measurements after various incubation periods (**h**). * Indicates a significant difference at *p* < 0.05 between Ridomil concentrations

Quantitative PCR analysis conducted to confirm the impact of the treatments on the absolute abundance of total bacteria showed no statistically significant differences among treatments in the total bacterial gene copy number (Fig. 4a). However, Ridomil application significantly increased the total gene copy number of *Pseudomonas* spp., which was consistent with the MiSeq data in which the Ridomil-treated soil exhibited the highest abundance of *Pseudomonas* compared to the other treatments (Figs. 3 and 4b).

The impact of different concentrations of Ridomil on the growth of two *Pseudomonas* species, *P. palleroniana* B2020 and *P. protegens* B59979 was tested using a 96-well plate assay (Fig. 4h). The results showed that the recommended dose of Ridomil (0.49 µl/L) had variable effects on the growth of the *Pseudomonas* species. *P. protegens* B59979 was sensitive to Ridomil treatment at all concentrations during the log phase and showed a significant reduction in the growth rate, whereas *P. palleroniana* B2020 showed no significant differences.

### Abundance of total fungi, *Trichoderma* spp., and *Pythium* spp

Analysis of the effects of the treatments on the absolute abundance revealed that Ridomil alone or SoilGard in combination with *Pythium* significantly (*p* < 0.05) reduced the gene copy number of total fungi compared to the control (Fig. 4c). The fungal gene copy number of the 18 S rRNA gene ranged from log 7.61 to 8.07 copies per gram of soil, with the highest recorded in the untreated control. SoilGard in combination with *Pythium* resulted in a gene copy number of 7.67 which was significantly lower than that of the control. Interestingly, the highest copy number of the *Trichoderma-*specific tef1 gene (> log 5) was observed in SoilGard-treated soil, compared to the untreated control or Ridomil-treated soil (Fig. 4d), but Ridomil without *Pythium had* the lowest tef1 gene copy number (log 4.45).

Our study found no statistically significant differences (*p* > 0.05) in the ITS gene copy number for *Pythium* species, *P. irregulare*,* P. ultimum* among treatments (Fig. 4e-g). The total *Pythium* gene copy number ranged from log 7.03 to 7.18 copies/g soil, and artificial inoculation with *Pythium* did not have a significant effect on its absolute abundance. Species specific gene copies/g soil for *P. irregulare* ranged from log 5.9 to 6.4 copies/g soil, and log 6.19 to 6.36 copies/g soil for *P. ultimum* (Fig. 4f, g).

### Effects of SoilGard and Ridomil on microbial community metabolic activities

**Fig. 5:**
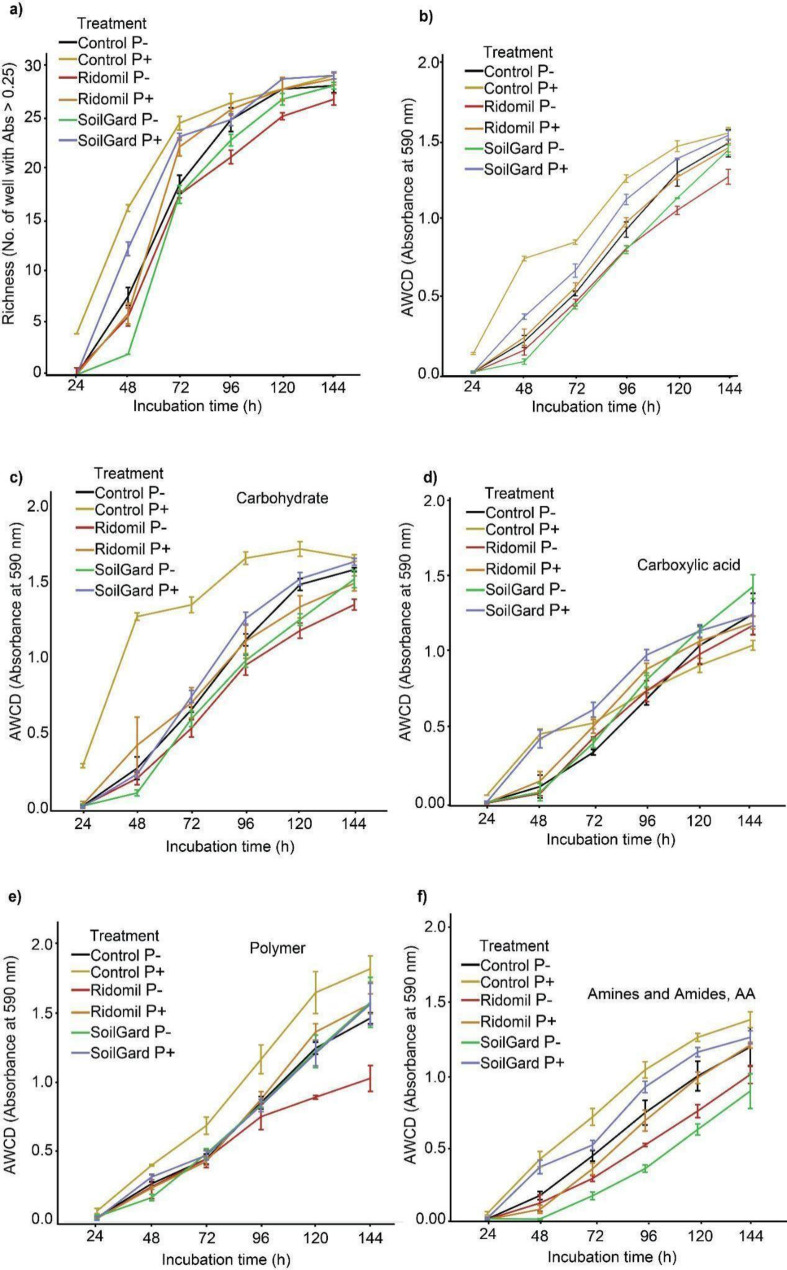
Utilization of different carbon sources by bacterial communities after soil treatment with SoilGard and Ridomil under carrot cultivation. Richness (**a**), average well color development (AWCD) of all carbon sources (**b**), carbohydrate (**c**), carboxylic (**d**), polymer (**e**), amines, amides, and amino acids (**f**)

In the investigation of changes in metabolic activities following Ridomil and SoilGard applications by examining the capacity of bacterial communities to metabolize 31 organic carbon sources, significant shifts were observed among treatments across all incubation periods. Overall C source utilization increased with longer incubation times (Table S5, Fig. 5a, b, c).

Among *Pythium*-inoculated samples, Ridomil showed the lowest richness and overall AWCD compared to untreated control during the early stages of incubation (24, 48 and 72 h) (Table S5, Fig. 5a, b). However, among *Pythium* non-inoculated samples, Ridomil’s significant negative impact on the metabolic activities of bacterial communities was observed during the later stages of incubation (96 to 144 h) (Table S5, Fig. 5a, b).

In *Pythium* inoculated soil, Ridomil and SoilGard treatments exhibited significantly reduced utilization of carbohydrates compared to control throughout the incubation periods, except after 144 h of incubation (Fig. 5c, Table S6). In the *Pythium* non-inoculated groups, a significant decrease in carbohydrate utilization was recorded at 120 and 144 h of incubation for Ridomil and at 120 h for SoilGard compared to the control. Contrastingly, in *Pythium*-inoculated samples, Ridomil showed lower metabolism of polymers and amines, amides and amino acids compared to the control at all incubation periods, except at 144 h.

**Fig. 6:**
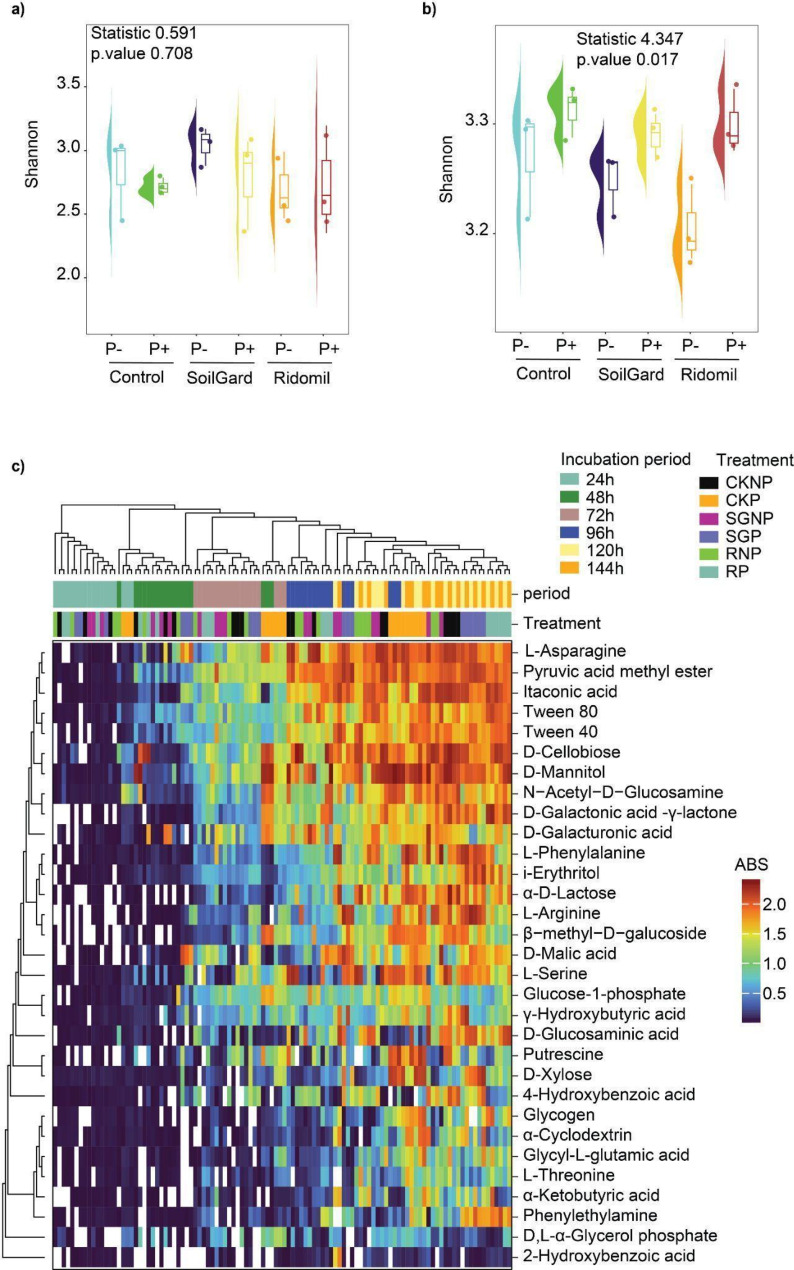
Variation in carbon utilization by bacterial communities in soil treated with SoilGard and Ridomil under carrot cultivation. Shannon diversity at 48 h (**a**) and 144 h (**b**) of incubation. Hierarchical clustered heat map of carbon sources utilization at different incubation periods (**c**)

Ridomil showed less utilization of carboxylic acid after 24 and 48 h incubation in the *Pythium-*inoculated soil compared to the control, but these differences were not observed in *Pythium* non-inoculated groups (Fig. 5d, Table S6). Ridomil and SoilGard treatments significantly reduced polymer utilization between 48 and 120 h of incubation in *Pythium-*inoculated groups compared to the control. However, in the *Pythium* non-inoculated groups such significant reduction was only observed in Ridomil after 120 and 144 h of incubation (Fig. 5e, Table S6). Notably, the effect of *Pythium* inoculation on carbon metabolism was more pronounced in the control treatment in carbohydrate, polymer and amines, amides and amino acids, while it was less significant in Ridomil and SoilGrad across all incubation periods (Fig. 5f, Table S6). Overall, the treatments and *Pythium* inoculation influenced carbon utilization as the *Pythium*-inoculated control had the highest metabolism of carbohydrate, polymer and amines, amides and amino acids carbon sources. The diversity of carbon utilization among treatments was statistically significant in the later stages of incubation than at the beginning (Fig. 6a, b, c).

### Carbon and nitrogen content

Our results showed no statistically significant differences (*p* > 0.05) in carbon, nitrogen content, or their ratio among treatments two weeks after treatment (Fig. S3 a-c). It is noteworthy that the Ridomil treatment, with and without *Pythium* inoculation, had relatively higher carbon (3.27, 3.13) and nitrogen (0.104, 0.09) contents compared to untreated control (2.52 carbon and 0.07 nitrogen) (Fig. S3 a and b).

## Discussion

SoilGard and Ridomil are effective strategies to combat carrot cavity spots caused by a *Pythium* spp. complex [13]. While the effects of several biological and chemical fungicides on soil bacterial communities have been widely studied, the specific impacts of SoilGard and Ridomil on bacterial communities and their metabolic activities remain poorly understood. Soil microbiomes are fundamental to key soil functions such as plant disease control, plant growth promotion, nutrient cycling and carbon cycling [2, 4]. Thus, in this study, we aimed to investigate the effects of Ridomil and SoilGard on the soil bacterial communities in pot grown carrots.

### Effects on bacterial diversity

Soil biodiversity is considered one of the key indicators of soil health [39] although there are conflicting studies that challenge this concept. The linkage between microbial diversity and soil health is poorly understood. Since microbial diversity is often highly correlated with soil pH, this may suggest that higher microbial diversity may not always indicate improved soil health [40]. Our results showed that the significant impacts of SoilGard and Ridomil on soil bacterial diversity were time-dependent. Ridomil had an immediate reduction of the soil bacterial diversity at the early stages of application compared to the SoilGard, which was less disruptive initially. However, SoilGard had delayed negative consequences when combined with *Pythium* inoculation, as observed at a later stage of soil sampling. These findings are consistent with previous studies that showed that chemical fungicides designed to target fungi or oomycetes can have negative effects on non-targeted bacterial diversity [41–47]. Although some studies indicated that *Trichoderma*-based biological control methods can significantly alter bacterial diversity [48–50], others reported no impact on non-target bacterial communities [51–53]. In addition, there are reports indicating that different types of fungicides have no effects on bacterial diversity [53–56], thus asserting that the judicious use of fungicides is safe for soil microbes [55], as these fungicides had the expected significant effects on targeted fungal communities [56, 57]. These contradictory findings indicate that there could be different factors dictating the outcomes of fungicide applications in various locations. Dose and frequency of fungicide application are key factors that influence the impact of both biological or chemical treatments on bacterial diversity [42, 46]. The significant reduction in bacterial diversity observed even after a single application of Ridomil and SoilGard highlights the need for further research to optimize their use and minimize effects on soil biology. The reduction in bacterial diversity shortly after two weeks of Ridomil application may be due to competition, selection and adaptation to the chemical fungicides [42, 43]. Importantly, these effects on non-target bacterial diversity may disrupt the natural assembly of soil microbial communities, potentially altering soil ecosystem function, therefore fungicides should be applied with caution [43]. Results from this study showing that Ridomil, particularly in the absence of *Pythium* inoculation, had a significant negative impact on total fungal abundance aligned with previous studies indicating that Ridomil negatively affects non-target fungal communities [13, 14]. The significant increase in the absolute abundance of *Trichoderma* spp. after SoilGard treatment was expected because SoilGard is 12% *T. virens*. However, the lack of a significant difference in the abundance of *Pythium* species between Ridomil-treated and non-treated soil warrants further investigation.

### Effects on bacterial community structure

The β-diversity analysis revealed that Ridomil-treated soil had a distinct bacterial community compared to the control, particularly in the enrichment of Proteobacteria, especially Gammaproteobacteria and Alphaproteobacteria. Similar to our findings, other studies have reported an increase in Proteobacteria abundance following the application of chemical fungicides [10, 54]. For example, the metalaxyl-mancozeb fungicide positively impacted the Proteobacteria population [53], which was attributed to the role of this bacteria in degrading chemicals. At the genus level, the abundance of *Pseudomonas* two weeks after the Ridomil application was confirmed by both MiSeq and qPCR data. Furthermore, our in vitro assay demonstrated that some *Pseudomonas* species can withstand high doses of Ridomil, thus strengthening our findings. However, an in-depth investigation with a larger number of *Pseudomonas* species is needed to validate this observation. In addition, several studies have reported the fungicide-resistant nature of *Pseudomonas* species to various fungicides [58]. Previous research noted that *Pseudomonas* was enriched in soils treated with the metalaxyl-mancozeb fungicide [44], and it showed high recovery in chlorothalonil and metalaxyl-m mixture applications [59]. Chakraborty [60] leveraged the Ridomil-resistant nature of *Pseudomonas* by combining it with Ridomil to effectively manage tomato damping-off caused by *Pythium aphanidermatum*. The compatibility of fungicides and *Pseudomonas* in managing plant diseases has been reported as an effective strategy in other studies [61, 62]. This suggested that further research is needed to fully understand the Ridomil-*Pseudomonas* interaction to further optimize their use in disease control. It is also noteworthy that *Devosia*, often associated with plant growth promotion and disease suppression [63–65] was enriched 12 weeks after Ridomil application. Although *Devosia* is known for detoxifying certain chemicals in the environment [66], its enrichment cannot be directly attributed to Ridomil decomposition because many factors can influence microbial dynamics over time, leading to an ecological shift and reduced competition, thus allowing *Devosia* to proliferate. However, the relative abundance of the second most dominant phyla, Bacteroidota, the class Bacteroidia and the genus *Flavobacterium*, as well as the phylum Bdellovibrionota, were highly reduced by Ridomil. Previous studies have identified *Flavobacterium* as a core taxon within the rhizosphere microbiome [67]. More importantly, reports have shown that *Flavobacterium* possesses the potential to suppress soil-borne plant diseases [68, 69]. Our findings align with previous reports that Bacteroidota are sensitive to synthetic chemical fungicides and are recognized as biological indicators of soil utilization [45, 70, 71]. As Bacteroidota are known for their role in organic matter degradation and nutrient cycling, further research is required to assess the ecological effects of Ridomil on soil bacterial communities in various field environments [71, 72]. Both Ridomil and SoilGard, when applied in the presence of *Pythium*, had negative effects on *Sphingomonas* populations two weeks after treatment. *Sphingomonas* are well-known for their beneficial roles in promoting plant growth and suppressing plant diseases [73–75]. SoilGard had a significant negative impact on the *Mycobacterium* population 12 weeks after application. There are hundreds of species of *Mycobacterium*, many of which are known to cause serious diseases in humans and animals [76]. Thus, the reduction of *Mycobacterium* populations in the soil due to SoilGard could have positive implications, as soil is a common source of infectious microbes. However, further research is needed to identify the specific species affected by SoilGard.

### Metabolic activities of bacterial communities in the soil samples

Soil microbes play a key role in driving nearly all ecosystem functions [77]. To assess the human impact on soil ecosystems, it is important to consider both taxonomic and functional diversity in biodiversity studies [78–81]. Community-level physiological profiling (CLPP) using Biolog Ecoplates is a commonly used method for monitoring the metabolic potential of microbial communities, based on the utilization of various carbon sources in 96-well plates [82–85]. The significant differences in substrate-level diversity among Ridomil and SoilGard-treated samples, based on carbon utilization in Biolog Ecoplates, indicates a shift in the metabolic activity of microbial communities [82]. It is noteworthy that Ridomil treatment exhibited the least taxonomic diversity, as well as the lowest richness and overall AWCD compared to untreated control, indicating that taxonomic diversity is a good indicator of changes in metabolic functions [79, 86]. However, high taxonomic diversity may not always correlate with functional diversity, as diverse microbes could share similar functional roles [80, 81]. This may be because rare species can disproportionately influence substrate diversity utilization [82]. The reduced metabolic activity in Ridomil-treated soils, particularly in polymer carbon sources, could be attributed to the direct toxic effects of Ridomil on bacterial communities, as we have observed that there are both Ridomil-sensitive and resistant *Pseudomonas* spp. The lower utilization of polymer carbon sources in Ridomil-treated soil may also be due to the dominance of *Pseudomonas* species in these soils, which have been shown to have limited ability to break down certain polymers [87].

## Conclusions

Our results showed that the impacts of Ridomil and SoilGard on bacterial communities, in terms of taxonomic diversity, were distinct and dependent on sampling time, and varied with *Pythium* inoculation. Ridomil had an immediate negative effect on bacterial taxonomic diversity, whereas SoilGard’s impact on bacterial diversity became apparent at a later time point after application, probably because the active ingredient in SoilGard, *Trichoderma*, takes time to establish and exert its effects. Given that both Ridomil and SoilGard altered the bacterial community composition, with only Ridomil enriching *Pseudomonas* spp., as confirmed by MiSeq and qPCR data, we can conclude that the effects of chemical and biological fungicides may vary with the treatment and are species-specific. In addition, both treatments have the potential to cause shifts in microbial metabolic activity that are in line with the taxonomic diversity. *Pythium* species can interact with the treatments to cause changes in the metabolic activity. For example, Ridomil in the absence of *Pythium* inoculation resulted in significantly lower richness and AWCD. This research highlights the distinct and time-dependent effects of biological and chemical fungicides applied at recommended doses on bacterial communities, supporting our hypothesis.

## Electronic supplementary material

Below is the link to the electronic supplementary material.

Supplementary Material 1

## Data Availability

The raw bacterial Illumina sequences of the 16 S rRNA gene used in this study have been deposited in the NCBI Sequence Read Archive (SRA) under the project number PRJNA1163035, with accession numbers ranging from SRX26135415 to SRX26135450.
