# Supplementary material for: A comparative study: impact of chemical and biological fungicides on soil bacterial communities
Source: Environ Microbiome. 2025 Apr 29;20:44. doi: 10.1186/s40793-025-00713-6 (PMC12042651; doi:10.1186/s40793-025-00713-6)
Supplement: Supplementary file 1 — Supplementary Material 1 [file 40793_2025_713_MOESM1_ESM.docx]

**A Comparative study: Impact of Chemical and Biological Fungicides on Soil Bacterial Community Dynamics**

Setu Bazie Tagele and Emma W. Gachomo*

Department of Microbiology and Plant Pathology, University of California Riverside, Riverside, CA 92507, U.S.A.

***Corresponding author**: Emma W. Gachomo (EWG); Email: emma.gachomo@ucr.edu

#

**Keywords:** Bacterial diversity, Eco-plate, Illumina MiSeq, Ridomil, SoilGard^TM^ 12G

#

#

# **Supplementary**


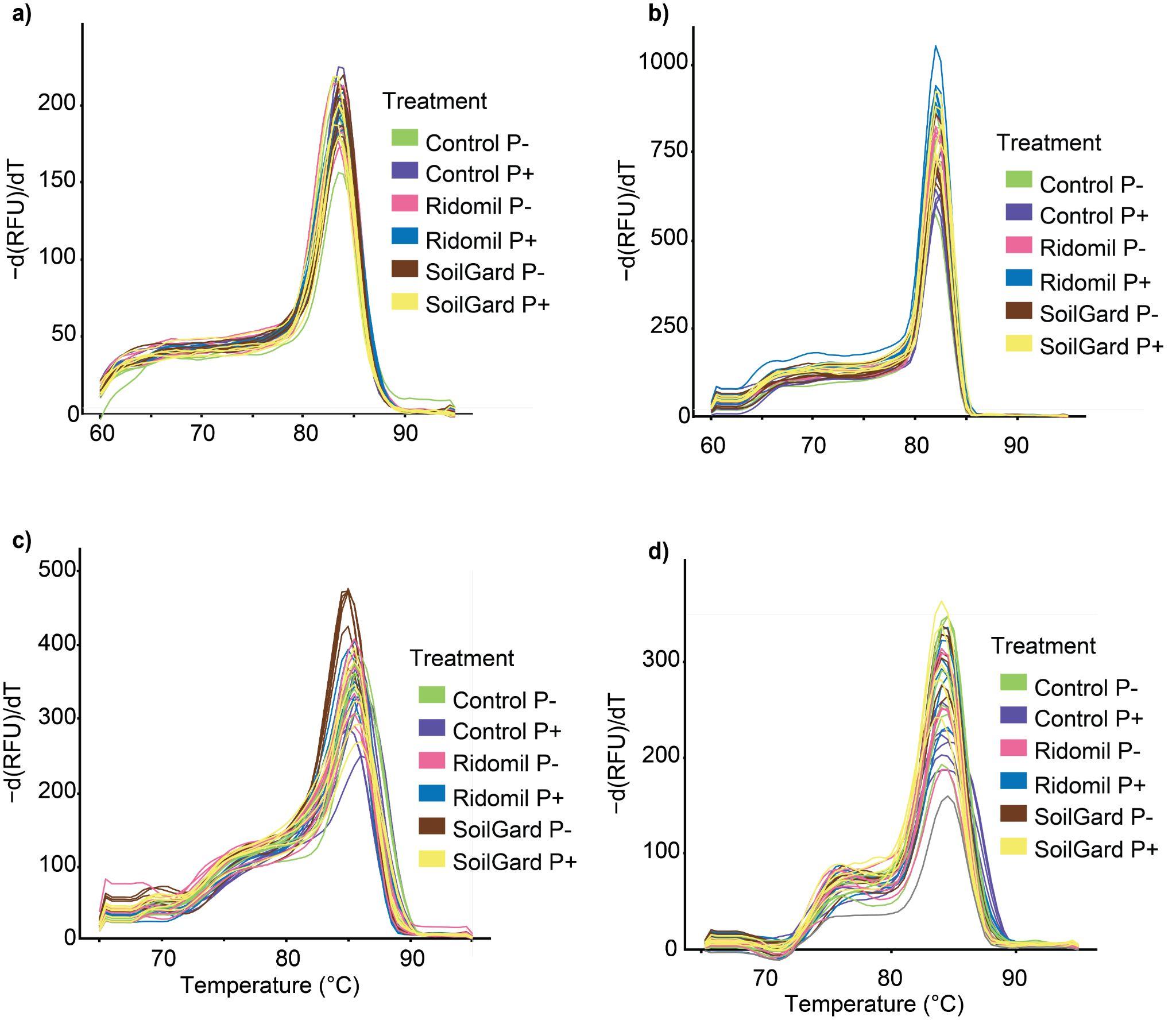


**Fig. S1**. Representative melting curves from qPCR-amplified products for total bacteria (a), *Pseudomonas* (b), total fungi (c), and *Pythium* species (d).

# **
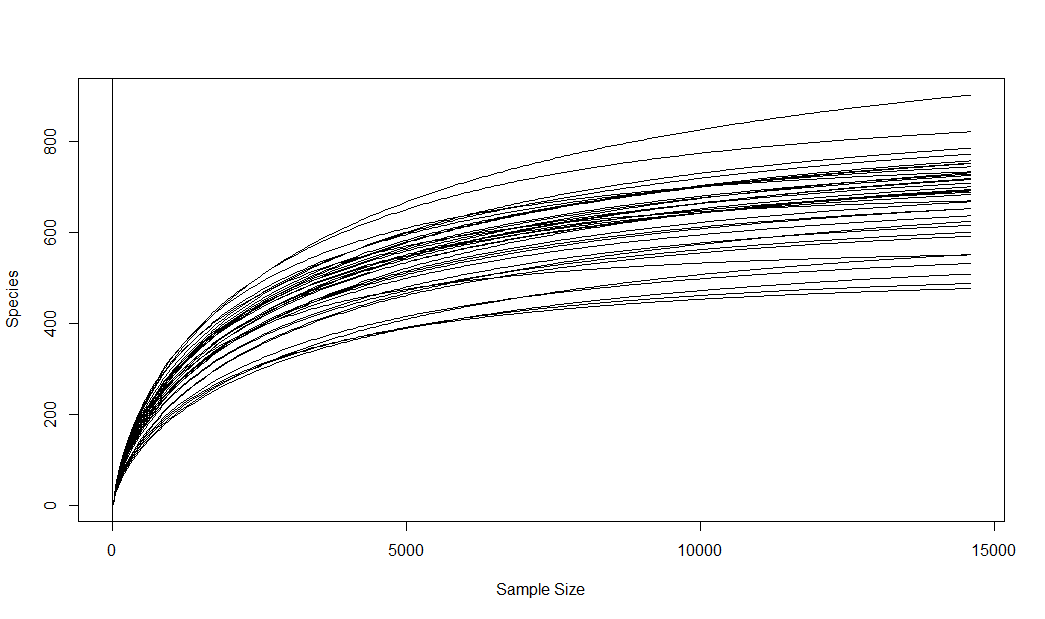
**

**Fig. S2: rarefaction curve analysis**

# **
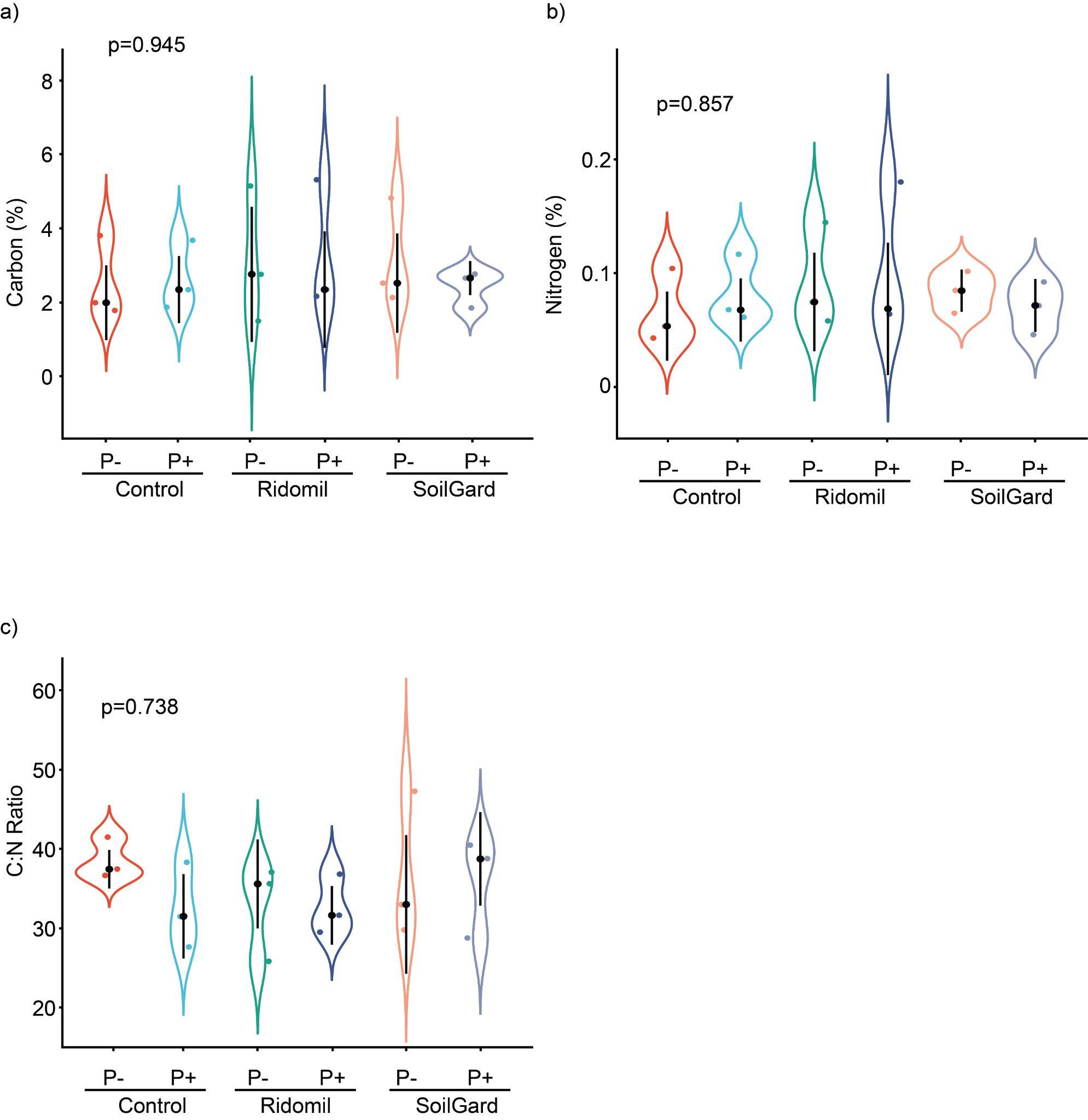
**

# **Fig. S3:** Violin plot showing the total carbon, nitrogen and the carbon:nitrogen ratio across different treatments two weeks after treatment

**Supplementary Table S1:** Primers used in quantifying bacterial, fungal and *Pythium* species

| Target | Forward 5'-3' | Reverse 5'-3' | Reference | Annealing temperature (°C) | Amplicon size (bp) |
| --- | --- | --- | --- | --- | --- |
| Total bacteria | GTGCCAGCMGCCGCGGTAA | GGACTACHVGGGTWTCTAAT | [(Caporaso et al. 2011; Katiraei et al. 2022)](https://paperpile.com/c/dmzRLA/a66R+e2Nk) | 55 | 252 |
| *Pseudomonas* species | ACTTTAAGTTGGGAGGAAGGG | ACACAGGAAATTCCACCACCC | [(Bergmark et al. 2012)](https://paperpile.com/c/dmzRLA/QULN) | 60 | 251 |
| Total fungi | AICCATTCAATCGGTAIT | CGATAACGAACGAGACCT | [(Chemidlin Prévost-Bouré et al. 2011)](https://paperpile.com/c/dmzRLA/u8Vf) | 50.1 | 390 |
| *Trichoderma* species | CCGTTTGATGCGGGGAGTCTA | GGCAAAGAGCAGCGAGGTA | [(Prabhakaran et al. 2015)](https://paperpile.com/c/dmzRLA/Gz2L) | 50 | 330 |
| *Pythium* species | TGCGGAAGGATCATTACCACAC | GCGTTCAAAATTTCGATGACTC | [(Boie et al. 2024)](https://paperpile.com/c/Bd0NPg/jLnY) | 60 | 421 |
| *Pythium irregulare* group I | GTATCGTCTTGGCGGAGTGG | TGCATAAACGAATATACCAACCGC | [(Schroeder et al. 2006)](https://paperpile.com/c/dmzRLA/kM2J) | 60 | 340 |
| *Pythium ultimum* | GACACTGGAACGGGAGTCAGC | AAAGGACTCGACAGATTCTCGATC | [(Schroeder et al. 2006)](https://paperpile.com/c/dmzRLA/kM2J) | 60 | 414 |

**Supplementary Table S2:** Effects of Ridomil and SoilGard at the phylum level

| Sampling time | Treatments | *Pythium* | Relative abundance (%) | | | | | |
| --- | --- | --- | --- | --- | --- | --- | --- | --- |
|  |  |  | Proteobacteria | Bacteroidota | Actinobacteriota | Chloroflexi | Bdellovibrionota | Patescibacteria |
| 2 weeks | Control | absent | 67.7 ± 3.13^bc^ | 11.7 ± 1.35^ab^ | 6.6 ± 1.21^bc^ | 3.4 ± 1.16 | 1.2 ± 0.08^a^ | 0.6 ± 0.13^b^ |
|  | Control | present | 64.4 ± 1.45^cd^ | 10.9 ± 1.27^ab^ | 9.7 ± 0.29^a^ | 2.5 ± 0.6 | 0.5 ± 0.2^bc^ | 2.2 ± 0.88^a^ |
|  | Ridomil | absent | 74.27^ab^ | 3.1 ± 0.33^d^ | 7.2 ± 1.37^abc^ | 3.7 ± 0.8 | 0.3 ± 0.02^c^ | 0.8 ± 0.14^b^ |
|  | Ridomil | present | 77.3 ± 2.36^a^ | 5.8 ± 1.5^cd^ | 4.8 ± 0.66^c^ | 2.8 ± 1.33 | 0.3 ± 0.08^c^ | 0.8 ± 0.11^b^ |
|  | SoilGard | absent | 60.5 ± 2.18^d^ | 14.5 ± 2.74^a^ | 9 ± 0.62^ab^ | 1.4 ± 0.29 | 1.1 ± 0.09^a^ | 2.6 ± 0.14^a^ |
|  | SoilGard | present | 71.3 ± 0.9^abc^ | 8.1 ± 0.63^bc^ | 5.8 ± 0.65^c^ | 2.8 ± 0.24 | 0.7 ± 0.07^b^ | 0.7 ± 0.03^b^ |
| p value |  |  | < 0.01 | < 0.01 | < 0.01 | 0.499 | < 0.001 | < 0.001 |
| 12 weeks | Control | absent | 59 ± 2.27 | 18.7 ± 4.31^ab^ | 10.4 ± 1.99^ab^ | 3.2 ± 0.46^b^ | 0.6 ± 0.07^a^ | 0.8 ± 0.16^b^ |
|  | Control | present | 59.5 ± 1.53 | 10.9 ± 1.68^bc^ | 14.1 ± 1.1^a^ | 3.3 ± 0.61^b^ | 0.6 ± 0.07^a^ | 0.8 ± 0.13^b^ |
|  | Ridomil | absent | 64.8 ± 2.04 | 8.8 ± 0.64^bc^ | 7 ± 0.63^bc^ | 4.7 ± 0.53^b^ | 0.3 ± 0.07^bc^ | 1.5 ± 0.53^ab^ |
|  | Ridomil | present | 60.8 ± 2.66 | 5.4 ± 0.49^c^ | 9.8 ± 1.92^abc^ | 10 ± 1.92^a^ | 0.3 ± 0.05^bc^ | 1.9 ± 0.35^a^ |
|  | SoilGard | absent | 58.1 ± 3.96 | 22.9 ± 3.45^a^ | 5.4 ± 0.49^c^ | 3 ± 0.31^b^ | 0.5 ± 0.13^ab^ | 1.4 ± 0.31^ab^ |
|  | SoilGard | present | 61.7 ± 2.77 | 18 ± 5.61^ab^ | 6.5 ± 1.38^bc^ | 5.5 ± 1.71^b^ | 0.2 ± 0.04^c^ | 0.5 ± 0.03^ab^ |
| p value |  |  | 0.557 | <0.05 | <0.01 | <0.01 | <0.01 | <0.05 |

**Supplementary Table S3:** Effects of Ridomil and SoilGard at the class level

| Sampling time | Treatments | *Pythium* | Relative abundance (%) | | | | | |  |
| --- | --- | --- | --- | --- | --- | --- | --- | --- | --- |
|  |  |  | Alphaproteobacteria | Gammaproteobacteria | Bacteroidia | Actinobacteria | Chloroflexia | Acidimicrobiia | Bacilli |
| 2 weeks | Control | absent | 33 ± 1.96^b^ | 34.7 ± 4.89^bc^ | 11.7 ± 1.35^ab^ | 3.3 ± 0.73 | 2.9 ± 1.15 | 2 ± 0.29^bcd^ | 2.3 ± 0.57 |
|  | Control | present | 42.8 ± 3.06^a^ | 21.4 ± 3.95^d^ | 10.9 ± 1.27^ab^ | 4.8 ± 0.55 | 1.7 ± 0.57 | 3 ± 0.51^a^ | 2.6 ± 0.55 |
|  | Ridomil | absent | 29.3 ± 2.43^bc^ | 44.7 ± 3.16^ab^ | 3.1 ± 0.33^d^ | 2.8 ± 0.66 | 3.1 ± 0.89 | 2.4 ± 0.12^abc^ | 3.9 ± 0.76 |
|  | Ridomil | present | 23.6 ± 1.74^c^ | 53.7 ± 2.49^a^ | 5.8 ± 1.5^cd^ | 2.7 ± 0.5 | 2.2 ± 1.14 | 1.4 ± 0.22^d^ | 3.1 ± 1.4 |
|  | SoilGard | absent | 35.9 ± 2.47^b^ | 24.5 ± 4.39^cd^ | 14.5 ± 2.74^a^ | 4.9 ± 0.7 | 0.6 ± 0.2 | 2.7 ± 0.14^ab^ | 3.4 ± 1.16 |
|  | SoilGard | present | 30.7 ± 1.23^bc^ | 40.5 ± 0.33^b^ | 8 ± 0.65^bc^ | 3.2 ± 0.66 | 2.1 ± 0.13 | 1.6 ± 0.16^cd^ | 1.9 ± 0.25 |
| p value |  |  | <0.01 | <0.01 | <0.01 | 0.09 | 0.317 | <0.05 | 0.629 |
| 12 weeks | Control | absent | 32.1 ± 3.98^c^ | 26.9 ± 1.97 | 18.6 ± 4.34^ab^ | 6.2 ± 1.57^ab^ | 2 ± 0.51^b^ | 2.2 ± 0.1 | 1.4 ± 0.15^bc^ |
|  | Control | present | 36.7 ± 2.75^bc^ | 22.7 ± 3.77 | 10.8 ± 1.69^bc^ | 8.4 ± 1^a^ | 2 ± 0.33^b^ | 3 ± 0.52 | 2.5 ± 0.29^a^ |
|  | Ridomil | absent | 48.3 ± 3.97^a^ | 16.5 ± 5.4 | 8.8 ± 0.64^bc^ | 3 ± 0.22^b^ | 3.4 ± 0.56^b^ | 2.1 ± 0.33 | 1.2 ± 0.06^bc^ |
|  | Ridomil | present | 44.8 ± 0.91^ab^ | 15.8 ± 1.76 | 5.4 ± 0.48^c^ | 5.9 ± 2.05^ab^ | 7.8 ± 2.21^a^ | 2.3 ± 0.14 | 1.8 ± 0.35^b^ |
|  | SoilGard | absent | 27.9 ± 2.32^c^ | 30 ± 2.68 | 22.8 ± 3.44^a^ | 2.9 ± 0.62^b^ | 1.1 ± 0.71^b^ | 1.7 ± 0.32 | 0.8 ± 0.14^c^ |
|  | SoilGard | present | 36.7 ± 5.63^bc^ | 24.9 ± 3.92 | 17.9 ± 5.6^ab^ | 2.5 ± 0.85^b^ | 4.2 ± 1.45^b^ | 2.5 ± 0.46 | 1.3 ± 0.12^bc^ |
| p value |  |  | <0.05 | 0.074 | <0.05 | <0.05 | <0.05 | 0.221 | <0.05 |

**Supplementary Table S4:** Effects of Ridomil and SoilGard at the genus level

| Sampling time | Treatments | *Pythium* | Relative abundance (%) | | | | | |
| --- | --- | --- | --- | --- | --- | --- | --- | --- |
|  |  |  | *Pseudomonas* | *Devosia* | *Flavobacterium* | *Sphingomonas* | *Mycobacterium* | *Brevundimonas* |
| 2 weeks | Control | absent | 13.3 ± 2.25^b^ | 3.2 ± 0.43^b^ | 5 ± 0.9^a^ | 4 ± 0.82^ab^ | 0.8 ± 0.17^b^ | 0.2 ± 0.04^bc^ |
|  | Control | present | 2.6 ± 0.99^b^ | 5.1 ± 0.92^a^ | 0.5 ± 0.17^b^ | 6.7 ± 1.96^a^ | 1.1 ± 0.04^b^ | 0.4 ± 0.09^a^ |
|  | Ridomil | absent | 33.9 ± 7.55^a^ | 3.2 ± 0.3^b^ | 0.2 ± 0.14^b^ | 1.5 ± 0.21^b^ | 1.4 ± 0.36^ab^ | 0.1 ± 0.03^c^ |
|  | Ridomil | present | 34.2 ± 6.16^a^ | 3.1 ± 0.6^b^ | 0.9 ± 0.71^b^ | 1.3 ± 0.19^b^ | 0.9 ± 0.05^b^ | 0.2 ± 0.02^c^ |
|  | SoilGard | absent | 2.9 ± 1.66^b^ | 4.3 ± 0.2^ab^ | 4.2 ± 1.4^a^ | 3.3 ± 0.33^b^ | 1.9 ± 0.1^a^ | 0.4 ± 0.1^a^ |
|  | SoilGard | present | 11.9 ± 1.46^b^ | 2.6 ± 0.46^b^ | 0.7 ± 0.43^b^ | 1.9 ± 0.35^b^ | 0.9 ± 0.13^b^ | 0.1 ± 0.05^c^ |
| p value |  |  | <0.001 | 0.05 | <0.01 | <0.01 | <0.01 | <0.05 |
| 12 weeks | Control | absent | 16.1 ± 2.16 | 3.7 ± 1.03^b^ | 13.9 ± 4.8^a^ | 1.2 ± 0.27 | 1.1 ± 0.21^bc^ | 1.1 ± 0.73 |
|  | Control | present | 8.1 ± 2.98 | 4.6 ± 0.32^b^ | 2.4 ± 0.64^bc^ | 2.9 ± 0.08 | 1.8 ± 0.23^a^ | 0.4 ± 0.12 |
|  | Ridomil | absent | 7.9 ± 3.4 | 9.8 ± 1.46^a^ | 0.9 ± 0.52^c^ | 1.2 ± 0.11 | 0.8 ± 0.05^cd^ | 0.3 ± 0.07 |
|  | Ridomil | present | 2.9 ± 1.83 | 7 ± 1.43^ab^ | 0.3 ± 0.13^c^ | 3.2 ± 1.98 | 1.5 ± 0.2^ab^ | 0.3 ± 0.04 |
|  | SoilGard | absent | 8.9 ± 3.73 | 4.6 ± 0.86^b^ | 11.2 ± 1.83^ab^ | 1.4 ± 0.21 | 0.5 ± 0.09^d^ | 0.4 ± 0.07 |
|  | SoilGard | present | 13.1 ± 0.62 | 7.3 ± 1.26^ab^ | 11.9 ± 5.79^ab^ | 0.8 ± 0.18 | 0.9 ± 0.25cd | 0.2 ± 0.1 |
| p value |  |  | 0.056 | <0.05 | <0.05 | 0.261 | <0.01 | 0.336 |

**Supplementary Table S5:** Biolog average well color development across and richness

| Incubation period (h) | Treatment | *Pythium* | Richness | AWCD |
| --- | --- | --- | --- | --- |
| 24 | Control | absent | 0.0 ± 0^b^ | 0.025 ± 0.01^b^ |
|  | Control | present | 4.0 ± 0^a^ | 0.145 ± 0.01^a^ |
|  | Ridomil | absent | 0.33 ± 0.34^b^ | 0.021 ± 0.01^b^ |
|  | Ridomil | present | 0.0 ± 0^b^ | 0.025 ± 0.01^b^ |
|  | SoilGard | absent | 0.0 ± 0^b^ | 0.026 ± 0.01^b^ |
|  | SoilGard | present | 0.0 ± 0^b^ | 0.025 ± 0.01^b^ |
|  | p value |  | < 0.001 | < 0.001 |
| 48 | Control | absent | 7.7 ± 0.89^c^ | 0.223 ± 0.04^c^ |
|  | Control | present | 16.3 ± 0.34^a^ | 0.758 ± 0.02^a^ |
|  | Ridomil | absent | 5.7 ± 0.89^c^ | 0.167 ± 0.04^cd^ |
|  | Ridomil | present | 6.0 ± 1^c^ | 0.249 ± 0.06^c^ |
|  | SoilGard | absent | 2.0 ± 0^d^ | 0.093 ± 0.02^d^ |
|  | SoilGard | present | 12.3 ± 0.67^b^ | 0.383 ± 0.02^b^ |
|  | p value |  | < 0.001 | < 0.001 |
| 72 | Control | absent | 18.7 ± 0.89^c^ | 0.533 ± 0.02^cd^ |
|  | Control | present | 24.7 ± 0.67^a^ | 0.864 ± 0.02^a^ |
|  | Ridomil | absent | 17.7 ± 0.34^c^ | 0.474 ± 0.03^de^ |
|  | Ridomil | present | 22.3 ± 0.89^b^ | 0.572 ± 0.03^c^ |
|  | SoilGard | absent | 17.7 ± 0.89^c^ | 0.450 ± 0.02^a^ |
|  | SoilGard | present | 23.3 ± 0.34^ab^ | 0.680 ± 0.05^b^ |
|  | p value |  | < 0.001 | < 0.001 |
| 96 | Control | absent | 25.0 ± 1.16^ab^ | 0.945 ± 0.05^c^ |
|  | Control | present | 26.7 ± 0.89^a^ | 1.273 ± 0.03^a^ |
|  | Ridomil | absent | 21.3 ± 0.67^c^ | 0.823 ± 0.02^d^ |
|  | Ridomil | present | 26.0 ± 0.58^a^ | 0.992± 0.03^c^ |
|  | SoilGard | absent | 23.0 ± 0.58^bc^ | 0.816 ± 0.03^d^ |
|  | SoilGard | present | 25.0 ± 0.58^ab^ | 1.139 ± 0.04^b^ |
|  | p value |  | < 0.01 | < 0.001 |
| 120 | Control | absent | 28.0 ± 1 | 1.311 ± 0.09^b^ |
|  | Control | present | 28.0 ± 1 | 1.483 ± 0.04^a^ |
|  | Ridomil | absent | 25.3 ± 0.34 | 1.070 ± 0.03^c^ |
|  | Ridomil | present | 28.0 ± 1.16 | 1.284 ± 0.02^b^ |
|  | SoilGard | absent | 27.0 ± 0.58 | 1.148 ± 0.01^c^ |
|  | SoilGard | present | 29.0 ± 0 | 1.403 ± 0.01^ab^ |
|  | p value |  | 0.088 | < 0.001 |
| 144 | Control | absent | 28.3 ± 0.67^ab^ | 1.503 ± 0.09^a^ |
|  | Control | present | 29.3 ± 0.34^a^ | 1.567 ± 0.04^a^ |
|  | Ridomil | absent | 27.0 ± 0.58^b^ | 1.286 ± 0.05^b^ |
|  | Ridomil | present | 29.0 ± 0.58^a^ | 1.473 ± 0.03^a^ |
|  | SoilGard | absent | 28.3 ± 0.34^ab^ | 1.458 ± 0.04^a^ |
|  | SoilGard | present | 29.3 ± 0.34^a^ | 1.553 ± 0.04^a^ |
|  | p value |  | 0.043 | 0.017 |

AWCD=over all average well color development across the 30 carbon sources; Richness=Number of wells showing Abs over 0.25 at 590 nm; CKNP= control without *Pythium*; CKP= control with *Pythium;* RNP = Ridomil without *Pythium*; RP= Ridomil with *Pythium;* SGNP= SoilGard without *Pythium*; SGP= SoilGard with *Pythium*

**Supplementary Table S6:** Biolog-carbon utilization by categories

| Incubation period (h) | Treatment | *Pythium* | Carbohydrate | Carboxylic acid | Polymer | Amines,Amides and Aminoacids |
| --- | --- | --- | --- | --- | --- | --- |
| 24 | Control | absent | 0.033 ± 0.01^b^ | 0.017 ± 0.01^b^ | 0.032 ± 0.01 | 0.015 ± 0.01^b^ |
|  | Control | present | 0.300 ± 0.02^a^ | 0.065 ± 0.02^a^ | 0.078 ± 0.01 | 0.054 ± 0.02^a^ |
|  | Ridomil | absent | 0.032 ± 0.02^b^ | 0.013 ± 0.02^b^ | 0.024 ± 0.01 | 0.012 ± 0.01^b^ |
|  | Ridomil | present | 0.042 ± 0.03^b^ | 0.014 ± 0.01^b^ | 0.024 ± 0.01 | 0.011 ± 0.01^b^ |
|  | SoilGard | absent | 0.033 ± 0.02^b^ | 0.018 ± 0.02^b^ | 0.037 ± 0.01 | 0.016 ± 0.01^b^ |
|  | SoilGard | present | 0.027 ± 0.01^b^ | 0.019 ± 0.02^b^ | 0.026 ± 0.01 | 0.020 ± 0.01^b^ |
|  | p value |  | < 0.001 | < 0.001 | 0.055 | < 0.05 |
| 48 | Control | absent | 0.283 ± 0.08^bc^ | 0.122 ± 0.02^b^ | 0.278 ± 0.08^bc^ | 0.176 ± 0.03^b^ |
|  | Control | present | 1.287 ± 0.03^a^ | 0.472 ± 0.01^a^ | 0.413 ± 0.04^a^ | 0.426 ± 0.06^a^ |
|  | Ridomil | absent | 0.216 ± 0.05^bc^ | 0.076 ± 0.09^b^ | 0.256 ± 0.02^bc^ | 0.124 ± 0.06^bc^ |
|  | Ridomil | present | 0.435 ± 0.19^b^ | 0.159 ± 0.02^b^ | 0.247 ± 0.07^bc^ | 0.081 ± 0.03^bc^ |
|  | SoilGard | absent | 0.119 ± 0.02^c^ | 0.085 ± 0.03^b^ | 0.177 ± 0.06^c^ | 0.015 ± 0.01^c^ |
|  | SoilGard | present | 0.244 ± 0.03^bc^ | 0.439 ± 0.02^a^ | 0.327 ± 0.06^ab^ | 0.372 ± 0.05^a^ |
|  | p value |  | < 0.001 | < 0.001 | 0.018 | < 0.001 |
| 72 | Control | absent | 0.670 ± 0.02^bc^ | 0.350 ± 0.04^d^ | 0.458 ± 0.02^b^ | 0.449 ± 0.04^bc^ |
|  | Control | present | 1.365 ± 0.06^a^ | 0.544± 0.07^ab^ | 0.702 ± 0.04^a^ | 0.716 ± 0.06^a^ |
|  | Ridomil | absent | 0.547 ± 0.06^c^ | 0.446 ± 0.07^bcd^ | 0.458 ± 0.01^b^ | 0.293 ± 0.03^d^ |
|  | Ridomil | present | 0.716 ± 0.11^bc^ | 0.522 ± 0.03^bc^ | 0.441 ± 0.05^b^ | 0.359 ± 0.05^cd^ |
|  | SoilGard | absent | 0.610 ± 0.06^bc^ | 0.411 ± 0.04^cd^ | 0.497 ± 0.04^b^ | 0.174 ± 0.03^e^ |
|  | SoilGard | present | 0.758 ± 0.05^b^ | 0.633 ± 0.04^a^ | 0.485 ± 0.05^b^ | 0.523 ± 0.04^b^ |
|  | p value |  | < 0.001 | < 0.01 | 0.015 | < 0.001 |
| 96 | Control | absent | 1.133 ± 0.04^bc^ | 0.704 ± 0.05^c^ | 0.869 ± 0.04^b^ | 0.745 ± 0.09^b^ |
|  | Control | present | 1.674 ± 0.05^a^ | 0.758 ± 0.11^c^ | 1.185± 0.02^a^ | 1.040 ± 0.06^a^ |
|  | Ridomil | absent | 0.966 ± 0.07^c^ | 0.757 ± 0.1^c^ | 0.767 ± 0.07^b^ | 0.523 ± 0.02^c^ |
|  | Ridomil | present | 1.125 ± 0.12^bc^ | 0.901 ± 0.06^ab^ | 0.894 ± 0.05^b^ | 0.691 ± 0.08^b^ |
|  | SoilGard | absent | 0.996 ± 0.05^c^ | 0.831 ± 0.03^bc^ | 0.855 ± 0.05^b^ | 0.361 ± 0.03^c^ |
|  | SoilGard | present | 1.274 ± 0.05^b^ | 0.996 ± 0.05^a^ | 0.849 ± 0.04^b^ | 0.923 ± 0.04^a^ |
|  | p value |  | < 0.001 | < 0.01 | 0.015 | < 0.001 |
| 120 | Control | absent | 1.500 ± 0.04^bc^ | 1.060 ± 0.05 | 1.267 ± 0.13^b^ | 0.998 ± 0.11^b^ |
|  | Control | present | 1.735 ± 0.05^a^ | 0.928 ± 0.16 | 1.673 ± 0.05^a^ | 1.264 ± 0.03^a^ |
|  | Ridomil | absent | 1.194 ± 0.05^d^ | 1.002 ± 0.02 | 0.909 ± 0.07^c^ | 0.757 ± 0.05^c^ |
|  | Ridomil | present | 1.352 ± 0.08^cd^ | 1.088 ± 0.06 | 1.386 ± 0.02^b^ | 0.986 ± 0.04^b^ |
|  | SoilGard | absent | 1.267 ± 0.04^d^ | 1.165 ± 0.12 | 1.244 ± 0.02^b^ | 0.630 ± 0.04^c^ |
|  | SoilGard | present | 1.537 ± 0.05^b^ | 1.156 ± 0.1 | 1.230 ± 0.05^b^ | 1.162 ± 0.04^a^ |
|  | p value |  | < 0.001 | 0.143 | < 0.01 | < 0.001 |
| 144 | Control | absent | 1.598 ± 0.02^abc^ | 1.269 ± 0.04 | 1.486 ± 0.14^a^ | 1.197 ± 0.14^ab^ |
|  | Control | present | 1.675 ± 0.03^a^ | 1.061 ± 0.1 | 1.846 ± 0.04^a^ | 1.385 ± 0.06^a^ |
|  | Ridomil | absent | 1.366± 0.04^d^ | 1.193 ± 0.1 | 1.046 ± 0.07^b^ | 1.008 ± 0.07^bc^ |
|  | Ridomil | present | 1.507 ± 0.05^c^ | 1.212 ± 0.08 | 1.591 ± 0.05^a^ | 1.210 ± 0.02^ab^ |
|  | SoilGard | absent | 1.536 ± 0.06^bc^ | 1.450 ± 0.19 | 1.602 ± 0.08^a^ | 0.894 ± 0.13^c^ |
|  | SoilGard | present | 1.650 ± 0.03^ab^ | 1.264 ± 0.16 | 1.588 ± 0.08^a^ | 1.264 ± 0.05^ab^ |
|  | p value |  | < 0.001 | 0.083 | < 0.01 | < 0.05 |

CKNP= control without *Pythium*; CKP= control with *Pythium;* RNP = Ridomil without *Pythium*; RP= Ridomil with *Pythium;* SGNP= SoilGard without *Pythium*; SGP= SoilGard with *Pythium*
